# Antimicrobial and Biocompatible Polycaprolactone and Copper Oxide Nanoparticle Wound Dressings against Methicillin-Resistant *Staphylococcus aureus*

**DOI:** 10.3390/nano10091692

**Published:** 2020-08-28

**Authors:** Jennifer Balcucho, Diana M. Narváez, Jinneth Lorena Castro-Mayorga

**Affiliations:** 1 Nanotechnology and Applied Microbiology Research Group (NANOBIOT), Department of Biological Sciences, University of the Andes, Bogotá 111711, Colombia; jt.balcucho298@uniandes.edu.co; 2 Human Genetics Laboratory, Department of Biological Sciences, University of the Andes, Bogotá 111711, Colombia; di-narva@uniandes.edu.co

**Keywords:** polycaprolactone, polymeric active films, copper nanoparticles, MRSA, antimicrobial activity

## Abstract

One of the major health problems linked to methicillin-resistant *Staphylococcus aureus* (MRSA) is severe diabetic foot ulcers (DFU), which are associated with hospital-acquired infections, lower limb amputations and emerging resistance to the current antibiotics. As an alternative, this work aims to develop a biodegradable and biocompatible material with antimicrobial capacity to prevent DFU. This was achieved by producing active polymeric films with metallic nanoparticles dispersed through a polycaprolactone (PCL) dressing. First, the antimicrobial activity of copper oxide nanoparticles (CuONPs) was tested by the microdilution method, selecting the lowest concentration that has an inhibitory effect on MRSA. Then, active PCL films were prepared and characterized in terms of their physicochemical properties, antimicrobial performance, cytotoxicity, genotoxicity and hemocompatibility. Active films had chemical and thermal properties like the ones without the antimicrobial agents, which was confirmed through FTIR, Thermogravimetric Analysis (TGA) and Differential Scanning Calorimetry (DSC) analysis. In relation to antimicrobial activity, active PCL films inhibited MRSA growth when treated with CuONPs at a concentration of 0.07% (*w*/*w*). After exposure to the active film extracts, human foreskin fibroblast cells (ATCC^®^ SCRC1041™) (HFF-1) exhibited a cell viability average above 80% for all treatments and no DNA damage was found. Finally, PCL films with 0.07% (*w*/*w*) CuONPs proved to be hemocompatible, and none of the films evaluated had red blood cell breakage greater than 5%, being within the acceptable limits established by the International Organization for Standardization ISO 10993-4:2002.

## 1. Introduction

*Staphylococcus aureus* constitutes a global health problem due to the variety and complexity of conditions caused in humans, commonly generating superficial skin conditions and lesions, but also being causative agents of pathologies such as pneumonia, endocarditis, infections of the nervous system, septic arthritis and generalized infections or sepsis. Likewise, the recent prevalence of pathogenic species that have developed resistance to the most frequently used antibiotics has been found [1]. One of the most important problems associated with *S. aureus* are severe diabetic foot infections, which are related to hospitalization in diabetic subjects and often lead to minor or major lower limb amputation. It has been found that diabetes mellitus affects around 9% of the adult population around the world [2]. Additionally, some studies establish that about 15% of diabetic patients are affected by diabetic foot ulcers at least once in their life and around 20% of clinical admissions worldwide are related to this type of ulcer [2].

Because of the increase in the number of microorganisms that are resistant to multiple antibiotics, many studies have been carried out to develop new antimicrobial agents that overcome the resistances of these microorganisms [3]. Metal nanoparticles are among the different compounds whose antimicrobial properties are being investigated, and have emerged as promising antibacterial agents [4,5]. Although several studies have been carried out to characterize silver nanoparticles and to propose mechanisms for its antimicrobial effectiveness [6,7,8], fewer studies have reported the antibacterial activity of copper nanoparticles and their potential as an antimicrobial agent [5,9,10,11]. It has been stated by some researchers that copper and copper oxide nanomaterials are easily mixed with polymeric solutions, in addition to the fact that they have more stable physical and chemical properties and are cheaper to synthetize, while a high degree of bioassimilation has been reported due to the fact that copper is a trace element in most living organisms [12,13,14,15]. With respect to nanoparticles of other metals such as Au and oxides of metals such as FeO and ZnO, these have also been studied as antimicrobial agents and biocides, but their disadvantages lie in their high synthesis costs and high instability and the few studies associated with biocompatibility, with the latter being used more in the manufacture of packaging with antimicrobial properties and as nanosensors [13,16,17]. 

Polycaprolactone (PCL) is a hydrophobic polyester that has been widely studied for different biomedical applications due to its high biocompatibility, miscibility with other polymers, unique rheological properties and controlled release of active compounds, which is directly related to its biodegradability [18,19]. Additionally, the biodegradability of PCL is associated to its molecular weight and crystallinity, since they affect the acid hydrolysis for which PCL polymeric chains breakdown into naturally occurring metabolites for further degradation and elimination from the body [20,21]. Therefore, PCL is a good candidate to be used as a structural component of dressings in direct contact with living tissues. 

Previous studies have reported the use of PCL for medical devices and drug delivery due to the controlled drug release associated to its high permeability in a wide range of pharmacological compounds, considerable biocompatibility with living tissues and biodegradability through the hydrolytic rupture of ester bonds [18,22]. In the case of metal nanoparticles, to date, several studies have investigated their cytotoxic hazard by using a wide range of experimental approaches [13]. Thus, thanks to advances in nanotechnology, it has been possible to generate materials with improved physicochemical properties and added antimicrobial properties [23], overcoming the obstacle of current treatments for diabetic foot ulcers (DFU), such as Primapore^®^, Mepora^®^ and cotton gauze whose purpose is limited to covering the lesion but does not protect it from infections by pathogens such as methicillin-resistant *Staphylococcus aureus* (MRSA) [24,25]. However, many studies in the field of metal nanoparticles have only focused on their cytotoxic effects when dispersed in liquid cultures, rather than studying their biocompatibility when immobilized onto a polymeric matrix, as was done in this research. To our knowledge, very few works have focused on the assembly of in vitro tests of the synthesized active polymeric materials, being almost null what has been reported with respect to the hemocompatibility and genotoxicity of films with immobilized metallic nanoparticles.

Based on the above analysis, this work is focused on the development of a biodegradable and biocompatible material with antimicrobial capacity to prevent diabetic foot infections associated with MRSA. The main objective of this research was to develop active films for biomedical applications with new or improved properties, such as efficient and prolonged antimicrobial activity, a lack of cytotoxicity and genotoxicity, and hemocompatibility. First, the antimicrobial activity of copper oxide nanoparticles (CuONPs) against the pathogen was evaluated in liquid cultures. Then, the polymeric films of PCL with the dispersed metal nanoparticles were prepared by a solvent-casting technique and their antimicrobial performance was assessed according to the Japanese Industrial Standard (JIS) Z 2801 (ISO 22196:2011). Finally, these polymeric films were characterized in terms of their biological and structural properties at the in vitro level. 

## 2. Materials and Methods 

### 2.1. Materials and Bacterial Strains

The MRSA strain used was supplied by the Microbial Research Center (CIMIC, University of the Andes, Bogotá, Colombia). Copper oxide nanoparticles (CuONPs) powder was supplied by Hefei Quantum Quelle Nano Science & Technology Co., Ltd. (Hefei, China). Polycaprolactone flakes (Sigma-Aldrich, San Luis, MO, USA, Average grade, Mn 80,000 g/mol), chloroform stabilized with ethanol (Technical grade), methanol (For analysis, ACS, ISO), 2.5 M NaCl, ethylenediaminetetraacetic acid (EDTA), tris(hydroxymethyl)aminomethane (Tris), dimethyl sulfoxide (DMSO), 1% Triton X-100 and NaOH were used throughout the experiments.

### 2.2. Effect of CuONPs on MRSA Growth

A volume of 10 µL of MRSA was transferred from a frozen stock (−80 °C in 15% (*w*/*v*) of glycerol) onto a Luria Bertani (LB) agar plate (Neogen, Lansing, MI, USA, supplement with 1.5% (*w*/*v*) agar) and incubated at 37 °C for 24 h. Afterwards, a colony from the plate culture was taken and transferred to a 100 mL Erlenmeyer containing 20 mL of LB broth and incubated at 37 °C for 24 h to 150 rpm. 

Sonication was used as dispersing agent in liquid cultures. For the above, an initial solution at 1000 ppm of CuONPs was sonicated with 0.97% (*w*/*v*) saline solution at 10 mL volume and under the following conditions: 47% power, 3 s ON and 6 s OFF for 90 s (VCX 130, Sonics & Materials Inc., New Town, CT, USA). Later, the sonicated solution was distributed into 100 mL Erlenmeyers, according to the final concentration of nanoparticles tested (20, 40, 60, 80, 100, 150, 200, 300, 350, 400, 500, 600 and 1000 ppm).

To identify the minimum inhibitory concentration (MIC) and the minimum bactericidal concentration (MBC), viable cell count experiments were carried out. In this case, bacterial liquid cultures were performed using 100 mL Erlenmeyer flasks with 20 mL of culture inoculated with 50 µL of MRSA and exposed to each nanoparticle concentration, which were then incubated at 37 °C and 150 rpm for 24 h. After that, LB agar plates were inoculated and incubated at 37 °C for 24 h. Finally, the nanoparticle concentration causing a bactericidal effect was selected based on the absence of colonies in the limit detection on the agar plates [26].

### 2.3. Preparation of Active Films

Polycaprolactone flakes were dissolved in a solution of chloroform (62.2% *w*/*v*) and butanol (37.8% *w*/*v*) under magnetic stirring for 30 min at 40 °C. Then, CuONP powder (0.05, 0.07 and 0.1 wt % respect to PCL mass) was added and stirred for 20 more min. Thereafter, the solutions were transferred to petri dishes and were dried to constant weight in an oven at 37 °C. Neat PCL films were used as controls for comparative purposes. 

### 2.4. Antimicrobial Activity of Active PCL Films 

Assays were done according to the Japanese Industrial Standard (JIS) Z 2801 (ISO 22196:2011, measurement of antibacterial activity on plastics and other nonporous surfaces) with some modifications. For the susceptibility test, a bacterial suspension with approximately 5 × 10^5^ Colony Forming Units per milliliter (CFU/mL) was applied over pieces of the active films of 3 × 3 cm and covered by an inert piece of Low-Density Polyethylene (LDPE) of 2.5 × 2.5 cm and 80 µm of thickness. After incubation for 24 h at room temperature and a relative humidity of at least 95%, bacteria were recovered with 0.97% (*w*/*v*) saline solution, and the viable cells were determined by the conventional plate count method. Films without nanoparticles were used as negative controls and an experimental setup was carried out with LDPE as an inert control, in order to check that the growth of MRSA was not affected by contact with PCL l. Three specimens of each sample were tested and the value of the bacterial reduction *(R)* was calculated by determining log_10_ (N_0_/N_t_), where N_0_ is the average of the number of viable cells of bacteria on PCL films without CuONPs after 24 h and N_t_ is the average of the number of viable cells of bacteria on the active films after 24 h. 

### 2.5. Characterization of the Active Films 

Samples of the active films were mounted on bevel sample holders with double-sided adhesive tape and sputtered with Au under a vacuum for morphology studies using a Scanning Electronic Microscope (SEM) at an accelerating voltage of 5 to 20 kV. The estimation of the average film thickness was done by means of micrometer measurement. To obtain an accurate estimation of the average diameter of the CuONPs in the active films, 200–300 measurements were done by means of the Adobe Photoshop software (CC 2019, Adobe Inc., MI, USA), from the SEM micrographs in their original magnification.

Thermal stability assays were done using Thermogravimetric Analysis (TGA) equipment. Samples were heated from 30 up to 1000 °C at 10 °C min^−1^ under nitrogen atmosphere. Derivative TGA curves (DTG) represent the weight loss rate as function of temperature and the temperature of maximum rate of degradation (Td). Tests were done in triplicate. Fourier-transform infrared spectroscopy (FTIR) spectra were obtained on a Nicolet 5700 FTIR spectrometer in the region from 4000 to 400 cm^−1^. The thermal properties of the films were evaluated using Differential Scanning Calorimetry (DSC) (Perkin-Elmer DSC 8000, Waltham, MA, USA). The analysis was carried out on approx. 3 mg of each sample at a heating rate of 10 °C min^−1^, from 0 °C to 200 °C, followed by a subsequent cooling down treatment to −50 °C. The DSC equipment was calibrated with indium as a standard and the slope of the thermograms was corrected by subtracting similar scans of an empty pan. Tests were done in triplicate.

### 2.6. Preparation of Active Extracts for Biological Activity

For the extracts, the active films were sterilized in 70% ethanol and exposed to UV overnight, and then washed with sterile PBS in triplicate, as proposed by ISO 109935 [27]. After that, extracts of the films were prepared by submerging a piece of 1 × 1 cm (0.219 ± 0.009 g) of each treatment (PCL + 0.05% CuONPs, PCL + 0.07% CuONPs, PCL + 0.1% CuONPs) in a sterile Eppendorf tube with 1 mL of Dulbecco’s Modified Eagle Medium (DMEM) (Sigma-Aldrich, San Luis, MO, USA) without serum for the cytotoxicity test, and 1 mL of Phosphate Buffered Saline (PBS) 1X for the genotoxicity test. In both cases, tubes were incubated at 37 °C for 48 h at 150 rpm in a humidified 5% CO_2_ atmosphere. One tube without the material was also incubated with DMEM or PBS 1X as a negative control.

### 2.7. Cell Culture

HFF-1 human foreskin fibroblast cells (ATCC^®^ SCRC1041™) were maintained in DMEM and supplemented with 15% of heat-inactivated fetal bovine serum (GIBCO) and 1% of penicillin/streptomycin (GIBCO). Cells were incubated at 37 °C in a humidified 5% CO_2_ atmosphere during two complete cell cycles (48 h) before treatment.

### 2.8. Cell Viability Assay

HFF-1 cell viability was determined by using the extracts of the PCL films with different CuONP concentrations in 96-well flat-bottomed plates; 1 × 10^5^ cells per mL were grown and exposed to 100 µL of the extracts in triplicate. Fibroblasts with DMEM but without serum were used as positive controls, and DMEM with 1% of Triton X-100 was used as a negative control. Blanks consisted of the extracts without cells for each formulation. Cell viability was studied with a 3-[4,5-dimethylthiazol-2-yl]-diphenyltetrazolium bromide (MTT) assay after 24 h of exposure to extracts by adding 10 µL of MTT (5 mg/mL) to each well and incubating the plate at 37 °C for 2 h. After this time, the medium was removed and 100 µL of DMSO was added to each well. Finally, formazan crystals were dissolved by agitation for 5 min and then, each well was analyzed in a BioRad micro plate reader at 595 nm, with a reference wavelength of 655 nm. The results were expressed as the percentage of living cells as calculated from MTT reduction, assuming the absorbance of control cells as 100%, and optical micrographs were taken in order to corroborate the results obtained in the cell viability assays [27,28].

### 2.9. Genotoxicity Assay

HFF-1 cells were incubated in 96-well flat-bottomed plates in duplicates at a concentration of 3 × 10^5^ cells per mL and grown for 24 h in supplemented DMEM. After incubation, cells were treated with 100 µL of PBS 1X extracts of the active PCL films. The negative control consisted of 100 µl of PBS 1X, and the positive control of H_2_O_2_ (80 µM in PBS 1X). Cells were incubated for 3 h at 37 °C and 5% CO_2_ atmosphere. After incubation, cells were washed, trypsinized and re-suspended in supplemented medium. Before the genotoxicity assay was performed, acute cytotoxicity was measured using the Trypan Blue dye exclusion assay. Briefly, a 50 µL aliquot was removed to treat cells with Trypan Blue (0.4%, Sigma) for 5 min. Treatments that exhibited a viability over 80% were used for genotoxicity by the Comet assay. 

The Comet assay was performed under alkaline conditions and the methodology used was adapted from that described by Sing et al. [29]. Briefly, an aliquot of 30 µL of each cell suspension was mixed with 270 µL of low-melting point agarose (0.65% *w*/*v*). Then, 100 µL of the previous suspension was spread on two precoated slides. When the agarose solidified at 4 °C for 8 min, a final layer of 0.65% low-melting-point agarose was applied to the slides and left at 4 °C for solidification. Slides were then left overnight in freshly prepared lysing solution at 4 °C (2.5 M NaCl, 100 mM ethylenediaminetetraacetic acid (EDTA), 10 mM tris(hydroxymethyl)aminomethane (Tris), 10% dimethyl sulfoxide (DMSO), 1% Triton X-100, pH 10). After lysis, slides were washed with PBS without calcium or magnesium and placed in alkaline buffer (300 mM NaOH, 1 mM EDTA, pH > 13, 4 °C) for 25 min. Electrophoresis was conducted at 25 V, 290 mA for 35 min at 4 °C. After that, the slides were treated with neutralizing buffer (0.4 M Tris, pH 7.5), dehydrated in methanol, and dried at room temperature. The slides were hydrated in cold deionized water and stained with Gel Green 3X (Biotium) for 5 min. Slides were washed in cold water and a coverslip was placed over the gel. Coded slides were examined with a Zeiss fluorescence microscope at 100x and using a 495-nm excitation filter and a 517-nm emission filter. A total of 100 cells were examined per treatment (25 cells per slide, 4 slides per treatment). The DNA damage was determined according to the percentage of DNA in the tail (% tail) using the CometScore software. 

### 2.10. Copper Content Release from the Active Films to the Extracts

To determine the total copper content within the extracts, high-resolution continuum source graphite furnace atomic absorption spectrometry (HRCS SS GFAAS) was done, as described by Feichtmeier et al. [30], with some modifications. A ContrAA700 spectrometer (Analytik Jena AG, Jena, Germany) was used, which is equipped with a graphite furnace atomization unit and an automated solid sampler (SSA 600) (Analytik Jena AG, Jena, Germany). For sampling, pyrolytic graphite-coated solid sampling tubes without dosing holes (Analytik Jena AG, no. 407-A81.303, Jena, Germany) were employed and samples were inserted into the graphite tubes. Argon with a purity of 99.999% (MTI, Neu-Ulm, Germany) was used as a purge and protective gas. Data evaluation was achieved with the ASpect software (CS 2.0.0, Analytik Jena AG, Jena, Germany) For atomic absorption, the Cu line at 324.754 nm was settled and the background was corrected by the iterative baseline correction (IBC) algorithm. The graphite furnace temperature program applied to the liquid samples of 40 µL each was the one presented in Table 1.

### 2.11. Hemocompatibility 

In order to evaluate the response of red blood cells to contact with the active films, human blood hemolysis tests were performed [31,32]. For this purpose, 25 mL of human blood was collected from an anonymous volunteer donor and stored in EDTA-coated K_2_ tubes to prevent the clotting of the sample. After this, the blood tubes were centrifuged at 1000 rpm for 5 min and then the levels of hematocytes were marked in order to discard the plasma in a container with water and hypochlorite. The discarded volume of plasma was completed with 0.9% (*w*/*v*) saline solution and the tubes were gently inverted several times to mix and centrifuge at 1000 rpm for 5 min. 

The washing step with saline was repeated 3 times for a final wash with PBS 1X, discarding the supernatant. Then, in a 96-well plate, dilutions of the erythrocytes were made with PBS 1X until reaching a concentration of 0.4% (v/v) per well and a final volume of 200 µL. According to the treatments, pieces of 5 mm^2^ of the neat PCL and PCL + 0.7% CuONPs films were placed in the wells in order to put them into contact with the erythrocytes. The negative control consisted of red blood cells with PBS 1X and the positive control consisted of red blood cells with Triton X-100. The seed plate was incubated with the different treatments at 37 °C and 5% CO_2_ for 1 h. Then, the contents of each well were centrifuged at 3000 rpm for 5 min and 75 µL of the supernatant of each treatment was recovered with a micropipette to read the absorbance at 450 nm. To calculate the percentage of hemolysis for each treatment, the following equation was performed:

(1) $$H\left(\%\right)=\frac{Abs\left(s\right)-Abs\left(nc\right)}{Abs\left(ps\right)-Abs\left(nc\right)}*100$$

where *Abs(s)* corresponds to the absorbance of the sample at 450 nm, while *Abs(nc*) to the absorbance of the negative control and *Abs(pc)* to that of the positive control, respectively. To obtain the average values of absorbance of the samples, triplicates per treatment were analyzed. 

To evaluate the coagulation of blood plasma in contact with the films tested, platelet aggregation trials were performed [31,33]. First, the human blood sample taken from an anonymous donor and stored in recovered K_2_ tubes treated with EDTA was centrifuged at 1000 rpm for 15 min at room temperature. Once the platelet-rich plasma was separated, 100 µL of it was taken for each treatment. In a 96-well plate, pieces of 5 mm^2^ of PCL and PCL + 0.07% CuONPs films were placed in the wells with plasma and left to incubate for 3 min at 37 °C. Oxidized PCL films with immobilized epinephrine were used as positive controls and PCL films with immobilized heparin were used as negative controls. After incubation, the films were removed from the wells and the absorbance of each treatment was read at 620 nm. The final values were reported in transmittance by working with triplicates per treatment. 

### 2.12. Statistical Analysis 

The statistical analyses were carried out by means of Prism 8 (8.4.3, GraphPad Software Inc., San Diego, CA, USA) and R Studio (1.2.1335, R Studio Inc., Boston, MA, USA) through the analysis of variance (ANOVA). Homogeneous sample groups were obtained by using Tukey and Dunnett’s honestly significant difference test (95% significance level).

## 3. Results and Discussion

### 3.1. Effect of CuONPs on MRSA Growth

**Figure 1:**
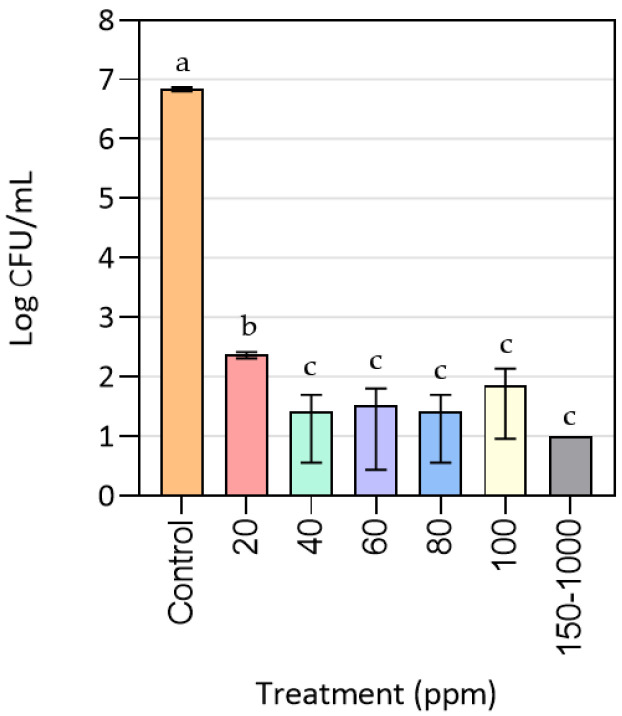
Antimicrobial activity of CuONPs against methicillin-resistant *Staphylococcus aureus* (MRSA). The 150–1000 bar represents the following concentrations: 150, 200, 300, 350, 400, 500, 600 and 1000 ppm. The detection limit was 10 CFU/mL. Mean values with different letters represent significant differences (*p* < 0.05) among the samples, as determined with a one-way analysis of variance (ANOVA) and Tukey’s multiple comparison tests.

Figure 1 exhibits the results for the plate count method after exposing MRSA cells to different concentrations of CuONPs. As can be seen, bacterial growth was completely inhibited at the range from 150 to 1000 ppm of CuONPs with an *R* = 6. This means that the MBC is equal to 150 ppm, as this was the minimum concentration at which bacterial death was achieved. In contrast, at concentrations lower than 100 ppm MRSA cells could proliferate. Even at a concentration of 20 ppm of nanoparticles cell growth was reduced at least in three logarithms (*R* = 3) when compared to the control treatment, indicating that this concentration would be considered as MIC since it inhibited bacterial proliferation, although it did not produce the death of the microorganism. From 40 to 100 ppm, the MRSA cell growth is similar, regardless of the nanoparticle concentration, which can be explained by CuONP aggregation throughout the cultivation period. The previous annotation was confirmed by the precipitates found at Erlenmeyer bottoms once cultures were taken from the shaker. This indicated that using sonication as the dispersing agent was not enough to obtain a homogeneous dispersion in the culture medium over time. 

As stated by Le Ouay and Stellacci [34], the antimicrobial action of metal nanoparticles highly depends on their surface reactivity. In this sense, smaller nanoparticles are desirable since they exhibit a stronger inhibitory effect due to their greater contact surface area and higher dissolution rate. This property allows them to interact more with the bacterial membranes and to move through them more easily. Moreover, these authors highlighted the importance of avoiding nanoparticle aggregation, as it reduces their effective specific surface and can lead to sedimentation. In addition, Kaur and Fanouraki [35] described that, in particle size analysis, it is important to disperse the sample using mechanical agitation, such as sonication, along with the physicochemical action of a dispersing agent, since these agents facilitate the separation of aggregated particles and provide the conditions to avoid flocculation or other interfering effects. 

However, after a previous literature review of the possible dispersing agents to be used, it was decided that sonication was the most viable option for the assays done in the present research. It has been reported by some authors that metallic nanoparticles tend to react in complex protein-rich media or with a wide variety of chemical compounds, due to their high reactivity. This is not desirable, since complexes of nanoparticles are formed, thus decreasing their antimicrobial activity, in addition to the fact that the presence of chemical compounds in the medium could affect the microbial culture itself [12,36,37,38].

Kruk et al. [39] established that the MIC for inhibitory assays of monodispersed CuONPs with an average size of 50 nm, against MRSA, was 3.75 ppm. Such a low MIC in this case could be due to the size of the nanoparticles used, being smaller than those used in this study. In relation to this, Ren et al. [12] found that the MIC for MRSA liquid cultures treated with CuONPs, within a size range of 22 to 94 nm, was 100 ppm, in accordance with the results found in the present research. Le Ouay and Stellacci [34] have explained that metal nanoparticles lose their antimicrobial activity as their size increases and once they aggregate, since the correct diffusion of the metal ions is truncated, so they are not able to reach the bacterial surface. 

According to some published studies, CuONPs exert an antimicrobial action through the release of metal ions that interact with the bacterial membranes and cellular components to finally induce cell death [5,12,40]. All authors explain that the interactions between those ions and the cell membranes, because of electrostatic forces, generate increased membrane permeability, the production of free radicals and reactive oxygen species (ROS), a loss of proton motive force, protein denaturation, the leakage of phosphate ions as well as cellular content and the disruption of DNA replication. Moreover, these studies establish that for the metal nanoparticles to exercise their antimicrobial activity, several aspects are important, such as the concentration of oxygen or the presence of an aerobic atmosphere needed to allow the oxidation of cellular macromolecules and components, and the generation of ROS. It was intuitively observed that culture agitation and the working volume have an influence on the growth rate of MRSA in liquid cultures and possibly affect the activity of nanoparticles, but measurements of dissolved oxygen and ROS generation are needed to prove this hypothesis. 

### 3.2. Antimicrobial Activity of Active PCL Films

**Figure 2:**
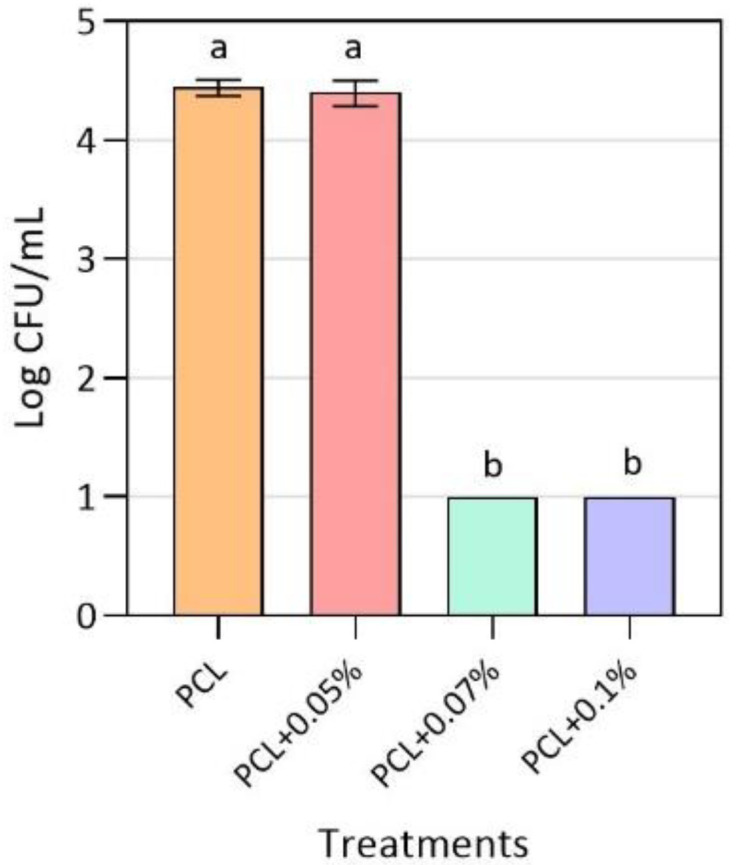
Antibacterial activity of neat polycaprolactone (PCL) films and active PCL films with different CuONPs concentrations against MRSA. The detection limit was 10 CFU/mL. Mean values with different letters represent significant differences (*p* < 0.05) among the samples, as determined with a one-way analysis of variance (ANOVA) and Tukey’s multiple comparison tests.

As shown in Figure 2, after 24 h of exposure to the active polymeric material, no viable cell counts of MRSA were recorded for the 0.07 and 0.1% (*w*/*w*) CuONPs concentrations. This results in a growth reduction by more than 4 log *(R = 4)* and establishes the MIC as 0.07% (*w*/*w*). For the 0.05% (*w*/*w*) CuONPs concentration, the final MRSA growth was similar to the one obtained for the control treatment, indicating that this concentration is too low to exert an inhibitory effect over the pathogenic strain. It should be noted that, for the antimicrobial activity assembly performed, a previous experiment was carried out in which the bacterial cells were exposed to the inert LDPE used, confirming that the growth of MRSA was not affected by it.

Previous studies published by Castro-Mayorga et al. [41] evidenced the total inhibition of the food pathogen *Listeria monocytogenes*, another Gram-positive bacteria, when exposed for 24 h to poly(3-hydroxybutyrate-co-3-hydroxyvalerate) (PHBV) films with concentrations of 0.05 and 0.1% (*w*/*w*) of the same CuONPs used in the present work. The previous result gives an insight of the use of nanomaterials in polymeric matrices for antimicrobial activity in a different field of application than the biomedical sphere. One of the greatest differences is that both polymeric matrices used present different hydrophobicity, which can affect the rate of release of metal ions to the environment by surface erosion, as the hydrophobicity of the material increases the rate of ion release. In this case, PHBV is more hydrophobic than PCL, as the former has an angle of contact of 103.61°, while the latter has an angle of 90° [33,42]. Additionally, PCL has been studied and implemented much more in the biomedical field, due to its high stability due to long-term degradation, its high biocompatibility and mechanical properties, such as superior rheological and viscoelastic properties, which make it easy to manufacture and manipulate into a wide range of three-dimensional platforms like cardiac grafts and as support for nerve and bone tissue regeneration [19,33,43,44].

Another study developed a polypropylene composite with embedded CuONPs of 40 nm at a concentration of 5% (*w*/*w*) for antimicrobial activity against *Escherichia coli* [45]. Even though it was concluded that, after 4 h of contact with the active films, 95% of the bacteria were killed, these results are not comparable to the ones obtained in the present research due to possible differences in the antimicrobial effect of metal nanoparticles in Gram-positive and Gram-negative bacteria [46]. According to the authors, there may be a difference in the antimicrobial activity of metal nanoparticles depending on the bacterial wall structure, explaining that the mechanism of action in Gram-positive bacteria occurs through nanoparticle internalization, while the inhibitory effect in Gram-negative bacteria is because of the release of copper ions and the interaction between the bacterial outer membrane. Similarly, a cotton material was coated with biosynthesized CuONPs with a size of 100–150 nm and exhibited an inhibition of 100% of *E. coli* and *S. aureus* after 48 h of incubation [47], while another study that used a cotton fabric coated with 1% (*w*/*w*) CuO by ultrasound radiation reported complete inhibition of *S.aureus* after 1 h of exposure [48]. In these cases, the inhibited strains were not drug-resistant ones and were more sensitive to the antimicrobial effect of metal nanoparticles for a longer exposure time, which could lead to an increase in the inhibition results. On the other hand, impregnating plated cellulose fibers with CuONPs at a concentration of 10% (*w*/*w*) resulted in a reduction of 99.5% in the viable colonies of MRSA after 1 h of exposure [13]. Again, this study tested a material with a higher copper oxide concentration, so a higher inhibition of six logarithms of the pathogen was observed. 

### 3.3. Films Characterization 

**Figure 3:**
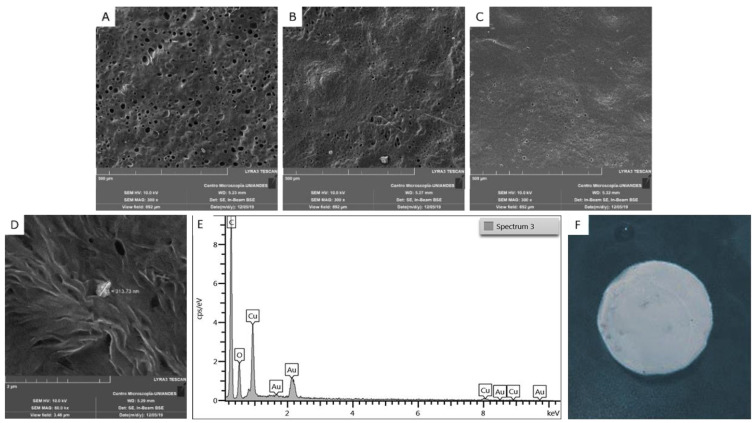
SEM micrograph of PCL films with copper oxide nanoparticles (**A**): PCL, (**B**): PCL + 0.1% CuONPs, (**C**): PCL + 0.07% CuONPs, (**D**): zoom-in image B, (**E**): EDS spectra for zoom-in image B, (**F**): Macroscopic aspect of PCL + 0.1% CuONP films.

The morphological features of the CuONPs used in this work are the ones previously reported by Castro Mayorga et al. [41] with a mean size of 191 nm, and the PCL films presented a mean thickness of 0.188 ± 0.073 µm. When analyzing the morphology of the films with dispersed CuONPs at different concentrations, aggregates of them with a mean size of 317 ± 4 nm were found, which were absent when comparing them with the control sample (Figure 3). Likewise, it was found that very few nanoparticles remained on the surface of the material, most CuONPs were inside the film and was not possible to visualize them by SEM. The previous phenomenon was due to the instability that these nanoparticles have, as a result of their size, as well as the viscosity of the polymer matrix when preparing the active films by the solvent-casting technique, which affects the nanoparticles’ dispersion through the material. Moreover, it can be observed that, on a microscopic level, the film without antimicrobial agent has a higher porosity than the active films, which could suggest that the presence of nanoparticles in the solution may affect the evaporation of the solvents during the synthesis of the polymeric films. In order to corroborate that the structures observed on the surface of the active films corresponding to the immobilized CuONPs, EDS tests were carried out, in which it was possible to identify the signal emitted by copper as the third most abundant element, after C and O. Regarding the macroscopic aspect of the films, no differences were observed between the neat PCL film and the active films.

**Figure 4:**
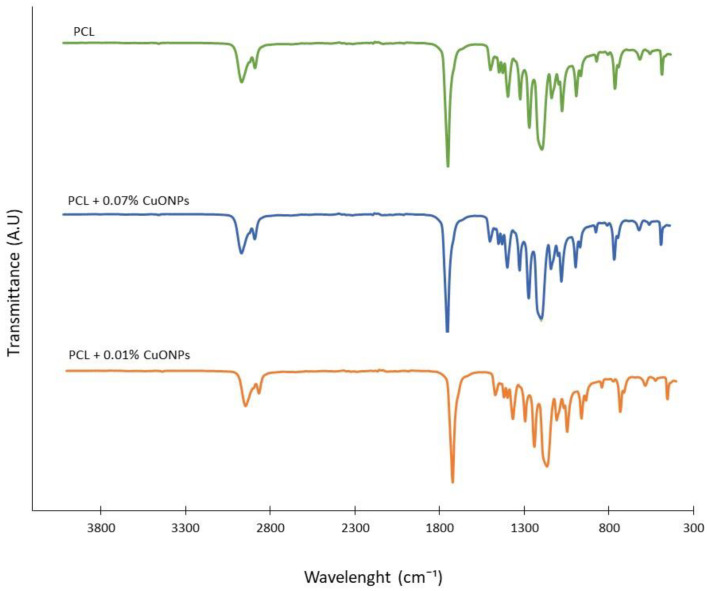
FTIR spectra neat PCL film and active PCL films with different CuONP concentrations.

As can be seen in Figure 4, active films with 0.05, 0.07 and 0.1% (*w*/*w*) of CuONPs displayed the same peaks in the FTIR spectra as films made out of neat PCL, which are the same values that have been reported to be characteristic of this polymer, being located at 3341, 1737, 1716, 850–1480 and 720 cm^−1^ [49]. It was determined that the concentrations of immobilized CuONPs in the active PCL films studied were not high enough to generate a change in the chemical features of the active materials. 

**Figure 5:**
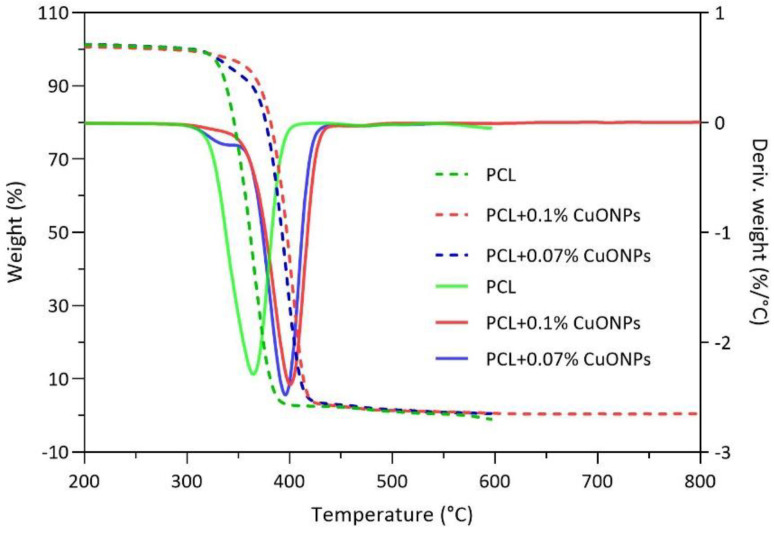
Thermal stability and degradation of active films with a heating rate of 10 °C min^−1^ for all the studied samples. The solid lines correspond to the weight (%), while the dashed lines correspond to the derivate of the weight (%/°C).

Thermal characterization was performed on samples of the polymer without nanoparticles and with 0.07% (*w*/*w*) CuONPs, since this concentration was established as the MBC. For the above, TGA analyses were conducted to evaluate the thermal degradation of neat and active PCL films when subjected to different temperatures with an established heating rate (Figure 5). As shown, the degradation of the neat PCL film started at 340 °C and continued up to 390 °C, the temperature range in which the PCL undergoes two consecutive mechanisms, the rupture of the polyester chains by pyrolysis followed by the formation of caprolactone, as described by Persenaire et al. [50]. Meanwhile, in the active films with 0.07% (*w*/*w*) of CuONPs, degradation started at around 370 °C and continued up to 420 °C, indicating an increase in the degradation temperature of around 30 °C when compared with the control sample. The previous results agree with the ones found by Ramos et al. [51], who found that when samples of neat PCL were exposed to a range of temperatures, a degradation peak was identified around 332–363 °C. In relation to the above, the study of Sivalingam and Madras [52] stated that the thermal degradation of this polymer, under inert atmosphere, occurs by the breakage of the polymeric chains in a random pattern due to ester pyrolysis, with the release of CO_2_, H_2_O and carboxylic acid. In addition, it is shown that thermal stability of the PCL matrix is affected by the metal nanoparticles since the neat PCL decomposition rate is the highest near 360 °C, whereas it was the highest at 400 °C for the active samples. Chrissafis and Bikiaris [53] describe that polymeric thermal stability is affected directly by the nature of the nanoparticles that are added to the final material and that, although nanomaterials do not affect the mechanism of degradation, they have an influence on the activation energies. The authors explained that the improvement in the thermal properties is related to the reduced permeability and diffusivity of oxygen and the degradation products.

### 3.4. Cell Viability Assays

**Figure 6:**
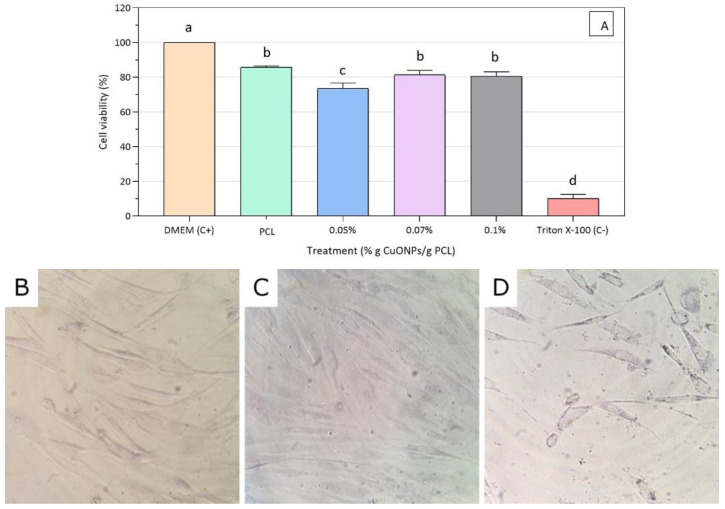
Viability results for HFF-1 cells exposed to neat PCL and active PCL films. (**A**): Viability test of the extracts of active PCL films on HFF-1 cells after 24 h of exposure, (**B**): HFF-1 with PCL + 0.07% CuONPs extract, (**C**): HFF-1 cells with Dulbecco’s Modified Eagle Medium (DMEM), (**D**): HFF-1 cells with Triton XCX-100; 40x magnification for the all three micrographs. Mean values with different letters represent significant differences (*p* < 0.05) among the samples as determined with a one-way analysis of variance (ANOVA) and Tukey’s multiple comparison tests.

A reduction of the MTT reagent was used as a cell viability indicator, since only active mitochondria contain dehydrogenase enzymes to cleave the tetrazolium ring, producing a color change from yellow to blue, allowing us not only to determine whether there is a cytotoxic effect in the HFF-1 cells, but also to elucidate the mechanisms by which CuONPs exert a toxic reaction [54]. After 24 h exposure to the active film extracts (PCL + 0.05% CuONPs, PCL + 0.07% CuONPs, PCL + 0.1% CuONPs), fibroblast cells exhibited a cell viability average above 80% for all treatments, except for PCL + 0.05% CuONPs when compared to the negative control (Figure 6A). HFF-1 cells treated with the PCL + 0.07% CuONPs film extract preserved the typical fibroblast morphology like the one observed in the positive control of cells exposed to DMEM media only (Figure 6B–D).

A possible explanation for the decrease in the viability of fibroblasts upon contact with the 0.05% (*w*/*w*) CuONPs active extract could be related to what was discussed above regarding the formation of nanoparticle aggregates due to instability in complex media. It is hypothesized that, probably, the higher the concentration of nanoparticles in the material, the less uniform the dispersion of the active agent in the medium is, directly affecting the inhibitory action of the nanoparticles by decreasing their cytotoxicity in the HFF-1 cells. 

In regard to the cytotoxic effects of dispersed nanoparticles, Bondarenko et al. [13] suggested that the median lowest concentration (L(E) C 50) value for CuONPs of around 200 nm is 1.1 × 10^4^ ppb for mammalian cells, being the lowest concentration of nanoparticles dissolved in water that kills half the population tested, while there was a reduction in human skin keratinocyte cell viability of 30% when exposed to CuONPs of 50 nm at a concentration of 2 × 10^4^ ppb for 24 h [55]. Likewise, Karlsson et al. [56] used the human lung epithelial cell line A549 to evaluate the cytotoxic effects of metal nanoparticles, arguing that CuONPs of less than 100 nm and at a concentration of 4 × 10^4^ ppb led to almost 100% cell death after 18 h of exposure. The authors explained that, since mammalian cell membranes are good barriers for most ions, nanoparticles may act as “Trojan-horse type carriers” by allowing the release of copper ions inside mammalian cells through acidic inclusions. 

All of the studies reviewed here support the hypothesis that the active PCL films synthetized in this research could be considered as safe superficial treatments for diabetic foot, since cell viability assays for the material seem to be in agreement with previous findings related to PCL itself, presenting a cell viability of more than 80% in almost all cases when conducting an approach related to mitochondrial damage. It is also important to point out that viability and cytotoxic results are strongly related to the cell type tested during the study, as Karlsson et al. [56] stated, which is relevant since the mammalian cells used during this research are human foreskin fibroblast HFF-1, a different human line to the ones used in the reviewed works. According to Lai et al. [57], different metal oxide nanoparticles had a different cytotoxic effect on human astrocytoma (U87) and fibroblast HFF-1, the latter being more resistant to titanium and zinc, but having the same viability decrease when exposed to magnesium nanoparticles. 

The research to date has tended to focus on the cytotoxic effect of metal nanoparticles dispersed on liquid media rather than their effect once immobilized into a polymeric matrix, as was done in the present work. Javed et al. [58] synthetized CuONPs that were capped by polyethylene glycol (PEG) and polyvinyl pyrrolidone (PVP) on the surface and after exposing brine shrimp (*Artemia salina*) to extracts of the films with different concentrations of nanoparticles (5 × 10^4^, 2.5 × 10^4^ and 1.3 × 10^4^ ppb) for the biocompatibility assay; all of the concentrations were found to be cytotoxic, with the highest one exhibiting almost 100% mortality. It should be noted that the material concentrations tested in this approach were considerably higher than the ones exhibit by the active films used in this study, which could explain the differences in the biocompatibility test results. Furthermore, the previous study conducted an in vivo assay to assess the biocompatibility of the material, whereas the present research relies on an in vitro approach, which suggests that the findings are not comparable to one another. Moreover, it has been found that aquatic organisms, such as algae, crustaceans and fish, tend to be more sensitive to metal NPs [13]. Due to the difference in the cytotoxicity of the metallic nanoparticles according to the exposed organism, the need to conduct in vivo tests to evaluate the biocompatibility of the active films synthesized in the present work becomes evident in order to give a better approximation of the effect that a longer exposure to these antimicrobial agents could have in humans.

In relation to the above and to evaluate the in vitro biocompatibility of PCL films, Serrano et al. [43] used L929 mouse fibroblasts and different aspects were studied, including mitochondrial function and cell adhesion and proliferation, stating that more than 24 h of culture is needed to reach a 100% cell attachment to the polymeric films and to stimulate thecell proliferation. The authors also found that mitochondrial activity was higher when fibroblasts were exposed to PCL films for 48 h. Similarly, several studies conducted by Lam and co-workers [59,60] have focused on exposing different animal models to PCL scaffolds and studying the short- and long-term biocompatibility, concluding that there are no adverse effects of these materials at a short-term exposure of 15 weeks and up to a long-term exposure time of 2 years. 

### 3.5. Genotoxic Effect of Active Extracts on HFF-1 Cells

In the Comet assay, apoptotic or necrotic cells may appear as false positives because cell death is associated with high levels of DNA damage; therefore, treatment with cytotoxic substances interfere with the identification of genotoxic agents [61]. In this study, all the active film extracts used show a cell viability above 80% after a 3 h treatment, as measured by the Trypan Blue assay. 

**Figure 7:**
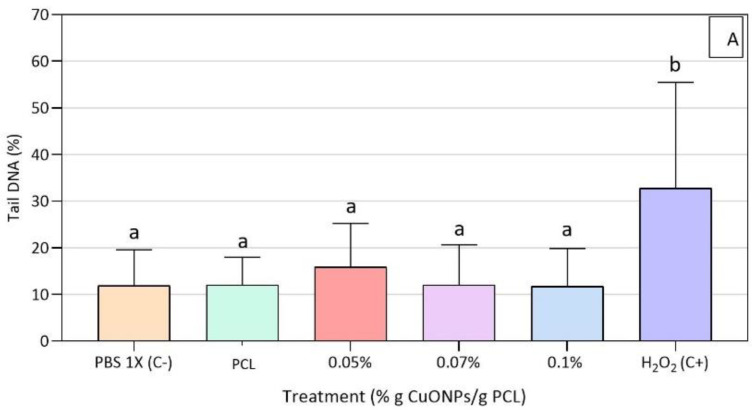

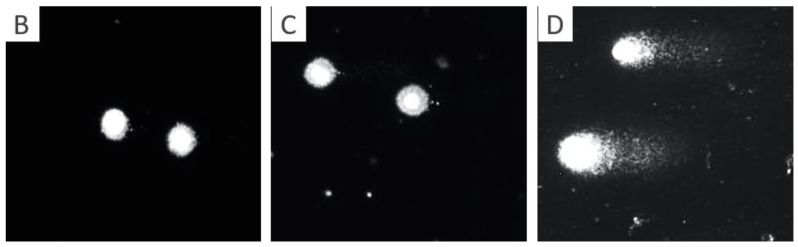
Genotoxicity results for HFF-1 cells exposed to neat PCL and active PCL films. (**A**): Genotoxicity assay on HFF-1 cells after 3 h of exposure to the active extracts, (**B**): HFF-1 cells with PCL + 0.07% CuONPs´ extract, (**C**): HFF-1 cells with DMEM, (**D**): HFF-1 cells with H_2_O_2_ (80 μM in PBS 1X). Mean values with different letters represent significant differences (*p* < 0.05) among the samples as determined with a one-way analysis of variance (ANOVA) and Tukey’s multiple comparison tests.

The induction of DNA damage was then measured by the alkaline Comet assay according to the percentage of DNA in the tail. No DNA damage was found for the active film extracts used and HFF-1 cells showed only significant differences for the positive control (Figure 7). 

In terms of DNA damage caused by CuONPs, the human lung epithelial cell line A549 evidenced DNA damage related to 40% of tail formation when cultured for 4 h with 8 × 10^4^ ppb of CuONPs of around 100 nm, concluding that DNA breaks were caused by oxidative lesions (16% tail) linked to an increase in intracellular ROS [56]. Similarly, human keratinocyte cells (HaCat) showed a DNA damage tail of around 20% after 24 h of treatment with 2 × 10^4^ ppb of CuONPs of 50 nm, while presenting a DNA damage tail of almost 30% when treated for 48 h and given that this damage was associated to oxidative stress, thanks to a decrease in glutathione and an increase in lipid peroxidation, catalase, and superoxide dismutase activity [55]. Additionally, the previous results agree with the potential mechanisms reported by Ahamed et al. [62], for which CuONPs exert genotoxic effects on human pulmonary epithelial cells (A549) in a dose-dependent manner. Regarding the possible mechanisms of the cytotoxicity of CuONPs in HaCat human keratinocytes and mouse embryonic fibroblasts, Luo et al. [63] argued that both an activation of Erk and a decrease in p53 protein levels regulate the toxic effect response of both cell lines to copper nanoparticles, with the first one being an intracellular signaling molecule that is involved in the regulation of mitosis and meiosis, and the second one a protein with a crucial role in the regulation of cell proliferation and in the cell’s response to stress stimuli. 

### 3.6. CuONPs Content in the Active Extracts

In order to determine the actual concentration of CuONPs that was released in the extracts used in the cell viability assays, these extracts were analyzed by HRCS GFAAS, as this is a technique that allows for the direct analysis of complex samples with very low detection limits and is widely used for the identification of agents of a metallic nature [57]. If it is assumed that the synthetized PCL films allow the release of all copper ions equivalent to the total immobilized CuO concentrations, the resulting active extracts to which the cells were exposed would have concentrations of 1.6 × 10^5^, 1.2 × 10^5^ and 8 × 10^4^ ppm, corresponding to the films with 0.1, 0.07 and 0.05% (*w*/*w*) CuO, respectively. These concentrations are higher than those reported as cytotoxic and genotoxic by the abovementioned articles in relation to the biocompatibility of metallic nanoparticles. 

Nonetheless, as can be seen in Table 2, when comparing the extracts of the active films without having been in contact with HFF-1 cells and after having been in contact with them, it was evident that the initial concentrations of copper ions to which these cells were exposed in the different tests were 7.84, 5.99 and 1.36 ppb, corresponding to the extracts of the films with CuONP concentrations of 0.1, 0.07 and 0.05% (*w*/*w*), respectively. These concentrations are significantly lower than the total content of copper ions in the synthetized films, as mentioned previously. Likewise, it was observed that, after 24 h of exposure of the cells to the active extracts, the residual concentration of copper in the samples was 4.80, 2.93 and 1.22 ppb, corresponding to the extracts of the active films of 0.1, 0.07 and 0.05% (*w*/*w*), respectively. As seen, some of the nanoparticles released into the media are absorbed by the cells, obtaining percentages of absorption of 38.78, 51.08 and 10.29% for the films of 0.1, 0.07 and 0.05% (*w*/*w*), respectively. However, the tests carried out to evaluate the viability of HFF-1 cells after being treated with these concentrations of copper ions released in the extracts showed that, in spite of the absorption of these ions, the concentrations used are neither cytotoxic nor genotoxic.

The results discussed above indicate that the copper ion concentrations to which the cells were exposed were considerably lower than the ones reported to be toxic in all the reviewed studies [13,55,56,57,58]. Moreover, the CuONPs used in the present study had a mean size of 191 nm, indicating that they are a lot bigger than the ones used in previous studies [13,55,56,57,58] and confirming the findings of Ingle et al. [64], who showed that the size is directly related to the toxic effect, as the smaller the size of the nanoparticles, the more toxic they are. As explained by Gunawan et al. [65] and Studer et al. [66], copper oxide nanoparticles can have different effects depending on the medium conditions in which they exist, with pH being one of the most important factors for their dissolution, along with amino species content. These authors reported that at an acidic pH or at high protein levels, CuONPs are dissolved, and copper ions are leached to the medium, so the cytotoxic effect is related to interactions between those metal ions and the cell membranes. Meanwhile, at neutral mediums or with a high content of dissolved salts, metal nanoparticles remain, and the cytotoxic effect is explained by an internalization and subsequent increase in ROS. As it stated in the present research, the assays were conducted on PBS, which is a neutral medium.

Some studies published by Borkow and co-workers [15,67] argued that copper-impregnated socks have been effectively used to heal skin lesions related to diabetic patients. This work established that when wound dressing with copper oxide was applied over inflicted wounds in genetically engineered diabetic mice, there was an increase in the expression of cell factors associated to tissue repair, as well as an increase in blood vessel formation and a decrease in the wound size. According to the authors, copper-impregnated socks release copper ions that can be absorbed through the skin, since copper is an essential trace element that induces skin regeneration by angiogenesis and the expression and stabilization of extracellular skin proteins, while it has been proven that the risk of adverse reactions due to dermal exposure to copper is extremely low. They hypothesized that one of the reasons why diabetic patients are prone to foot skin pathologies is due to low local copper levels, which are linked to the high concentration of glucose in the blood. Therefore, the need to conduct further studies implementing 3D models and in vivo analysis in order to really determine the effect of the contact of active PCL films with epithelial tissues and living organisms in general becomes evident. 

As was mentioned before, PCL was used in this study as an immobilization matrix for CuONPs since it has been reported to have several advantages over other biocompatible polymers currently used as wound dressing materials for DFU [25]. For example, cellulose, despite aiding in the proliferation of fibroblasts, creates problems with excess exudates in covered lesions [68], while chitosan presents difficulties during the dissolution process for the addition of agents [69] and collagen-based dressings usually require a second dressing to add structure to the material [70]. PCL has been recognized by the Food and Drug Administration Agency (FDA) as a safe material to be used for the release of therapeutic agents and for sutures, and its versatility has been reported when preparing solutions and working with different techniques, while promoting adhesion and cell proliferation on the generated material [25]. Finally, although one of its disadvantages has been described as its lack of antimicrobial activity, in the present work, we worked on this aspect by immobilizing CuONPs in the synthesized films. 

It should be noted that the tests carried out in this work are useful to verify that the polymeric matrix and the selected antimicrobial agent were adequate for the inhibition of the growth of MRSA, in order to continue with experimental tests that will allow us to improve the synthesis technique used, in this case going from generating active films of PCL by solvent casting to synthesizing nanofibers of PCL with CuONPs by electrospinning, since it has been reported that, with this technique, it is possible to generate porous materials that increase the biocompatibility of the material and the improvement of the treated lesion [19].

### 3.7. Hemocompatibility Assays 

In the biomedical field, it is important to develop materials that are proven to be hemocompatible, because blood is usually the physiological fluid with which they will have direct contact. Additionally, it has been established that the formation of blood clots on superficial biomedical materials are beneficial and desirable, since platelet aggregation will contribute to the wound healing process due to the release of growth factors and chemical signals to guide repair cells [71,72]. Therefore, in order to evaluate the behavior of the blood upon contact with the active PCL films, hemolysis and platelet aggregation tests were performed in vitro. Table 3 presents the hemolysis data obtained, in which it can be seen that both the PCL control films and the active films with 0.07% (*w*/*w*) CuONPs prove to be hemocompatible, presenting hemolysis values similar to saline and PBS, which are used as negative controls and are commonly used solutions when working with erythrocytes due to their stability under such conditions.

**Figure 8:**
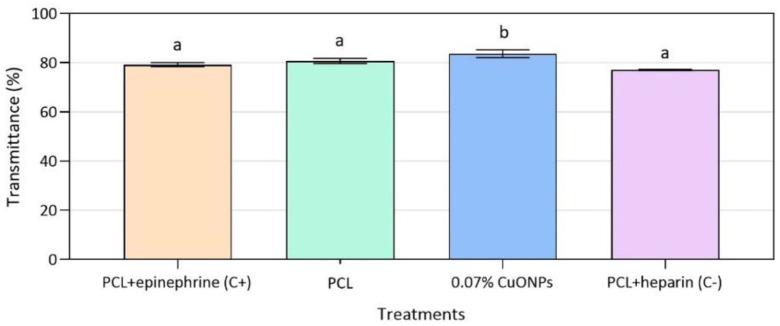
Platelet aggregation of human plasma on active PCL and neat PCL films after 3 min exposure. Mean values with different letters represent significant differences (*p* < 0.05) among the samples as determined with a one-way analysis of variance (ANOVA) and Tukey’s multiple comparison tests.

Likewise, none of the films evaluated had a red blood cell breakage greater than 5%, being within the acceptable limits established by ISO 10993-4:2002 [27]. In relation to platelet aggregation, both control films and films with CuONPs presented similar values of transmittance when compared to those presented by the positive control with immobilized epinephrine (Figure 8). It is important to emphasize that it was not possible to obtain adequate controls of the PCL film with epinephrine or heparin immobilized on the surface, as there were problems with the stabilization of these molecules. Therefore, it is necessary to continue working on the process of immobilization of biological molecules on this polymeric material.

Similarly, Padalhin et al. [33] carried out hemocompatibility tests for films of PCL mixtures with other biocompatible components in order to evaluate their possible use in bone tissue regeneration, reporting hemolysis values of less than 5% for all PCL mixtures, including the control of neat PCL, while a higher platelet aggregation on the surface of the films was achieved with the most hydrophobic membranes made of neat PCL and a mixture of PCL and poly (lactic-co-glycolic acid) (PCL/PLGA). The authors explain that the formation of platelet clots is directly related to the hydrophobicity of the material, establishing that the more hydrophilic the material, the more it tends to absorb proteins, which could reduce the deposition of platelets on its surface. Likewise, Li et al. [44] showed that increasing PEG content in polymeric films decreases platelet aggregation on the surface of the material due to decreased hydrophobicity, while neat PCL films show platelet aggregation with a pseudopod appearance observed by SEM, indicating the activation of blood clotting. 

## 4. Conclusions

Active PCL films synthetized in this research could be considered as safe superficial treatment for diabetic foot ulcers, presenting similar properties to previously accepted biocompatible materials used in the biomedical field. The active films achieved a methicillin-resistant *Staphylococcus aureus* growth inhibition of more than four logarithms at a concentration of 0.07% (*w*/*w*) CuONPs, while presenting stable thermal characteristics, as well as viability above 80% without DNA damage to treated HFF-1 cells. Additionally, the synthetized materials proved to be hemocompatible, since none the films evaluated had a red blood cell breakage greater than 5%, being within the acceptable limits established by ISO 10993-4:2002. Future in vivo biocompatibility tests are required in order to evaluate the response of complex organisms to the synthesized active PCL films.

## Figures and Tables

**Table 1:** Parameters for graphite furnace program for direct detection of copper oxide nanoparticles (CuONPs) in active extract samples by means of high-resolution continuum source graphite furnace atomic absorption spectrometry (HRCS SS GFAAS).

Step	Temperature (°C)	Heating Rate (°C/s)	Holding Time (s)
Drying I	90	3	20
Drying II	110	5	10
Pyrolysis I	350	50	20
Pyrolysis II	1100	300	10
Gas adaptation	1100	0	5
Atomization	2000	1500	4
Cleaning	2450	500	4

**Table 2:** HRCS GFAAS analysis of extracts of active PCL films with different concentrations of CuONPs.

Samples	Cu+ (ppb)
PCL + 0.1% CuONPs	7.84 ± 2.79 ^abd^
PCL + 0.07% CuONPs	5.99 ± 1.37 ^bde^
PCL + 0.05% CuONPs	1.36 ± 0.47 ^cef^
PCL + 0.1% CuONPs + HFF-1 cells	4.80 ± 0.20 ^de^
PCL + 0.07% CuONPs + HFF-1 cells	2.93 ± 0.14 ^ef^
PCL + 0.05% CuONPs + HFF-1 cells	1.22 ± 0.19 ^f^

Standard deviation is calculated from three measures. Mean values with different superscript letters in the same column represent significant differences (*p* < 0.05) among the samples according to ANOVA and Tukey’s multiple comparison tests.

**Table 3:** Hemolysis caused by neat PCL films and active PCL films.

Samples	H (%)
PCL	0.254 ± 0.096 ^a^
PCL + 0.07% CuONPs	0.263 ± 0.101 ^a^
Saline solution 0.9% (*w*/*v*)	0.247 ± 0.095 ^a^
Distilled water	8.487 ± 1.244 ^b^

Mean values with different superscript letters in the same column represent significant differences (*p* < 0.05) among the samples according to ANOVA and Tukey’s multiple comparison tests.
